# Quality of Life Amongst Multidrug-Resistant TB Patients: An Exploratory Study About Distributive Dimensions and Interactions

**DOI:** 10.7759/cureus.29389

**Published:** 2022-09-21

**Authors:** Shweta Sharma, Subba Krishna N, Arun Kokane, Abhijit P Pakhare, Mohammed M Nawaz, Ankur Joshi

**Affiliations:** 1 Community and Family Medicine, All India Institute of Medical Sciences, Bhopal, IND; 2 Community and Family Medicine, All India Institute of Medical Sciences, Guwahati, IND; 3 Community and Family medicine, All India Institute of Medical Sciences, Bhopal, IND

**Keywords:** multidrug-resistant tb, who-qol-bref, psychometric, national tuberculosis elimination program, quality of life (qol)

## Abstract

Background: Multidrug-resistant (MDR)-TB has emerged as a major challenge to eliminate TB as envisioned at policy level. Distinctive traits associated with the disease such as physical, psychosocial and environmental dimensions may influence the treatment outcome in both directions. Quality of life (QoL) indicators may capture these traits distinctively.

Objective: To quantify the differential effect of MDR-TB on specific QoL domains, their distributions across the strata and to check for possible interactions.

Method: This community-based cross-sectional study was conducted on 98 MDR-TB patients registered in the calendar year 2017 under National Tuberculosis Elimination Programme (NTEP) in a central Indian district using the WHO-QoL BREF Scale by patients in their vicinity. The transformed domain scores were descriptively summarized, stratified and exploratory visualised. Likert mapping for each item was done. A two-way ANOVA test was applied to check differences across strata and interaction effects were calculated.

Result: Participants perceived a higher QoL in the social domain (median score 69, IQR 56-75) while the psychological health domain (median 31 IQR 20.5-44) was professed as most negotiated by disease. More than 50% of participants were found to be dissatisfied with their assumed physical status in item-wise analysis. A statistically significant interaction (p=0.008) was detected with education strata to income tertile most evident in the physical domain while psychological domain alone (p=0.017) without significant interaction with treatment duration (p=0.316) was associated with the type of TB. Overall QoL scores were tilted in favour of an urban setting, male gender and towards a relatively younger population.

Conclusion: The overall deficits in QoL are evident in the study, per se in the psychological and physical domains. Moreover there is an inequitable distribution of these scores as revealed in the study. Inclusion of an additional parameter of periodical QoL assessment may thus predict the outcome at individual level and may address this inequity at policy level.

## Introduction

The emergence of drug resistance against Mycobacterium tuberculosis has been a growing threat to public health [1-2]. Tuberculosis (TB) epidemic in recent times has temporally been associated with social determinants like rapid urbanisation, poor nutrition, socioeconomic and living condition which have affected both the emergence/progression of the disease as well as issues related to treatment accessibility and adherence [3-5]. The length, complexity and adverse effects associated with therapy have intricated already complex phenomena [6]. A successful outcome not only depends on the allocation to the directly observed treatment, short-course (DOTS) therapy but also on behavioural interventions, psychosocial environment that become a necessary element for securing treatment adherence and thereby decreasing drug resistance [7]. These all factors in unison especially in context of multidrug-resistant (MDR)-TB culminates further into higher psychosocial comorbidities and economic crisis to maintain decent family life [8].

Philosophically health is conceptualised as the state beyond the conventional indicators of morbidity and mortality and adds quality component to it [9]. By mentioning quality at this juncture, it assigns a notion of the perceptual wellbeing of an individual affected by disease under inquiry [10-11]. As there is limited evidence regarding health-related quality of life (HRQoL) among drug-resistant TB patients, our study was an attempt to analyse the impact of the illness on the QoL [12].

This multidimensional quality component in MDR-TB requires an equally sensitive and wide-ranging tool. This can be measured with the help of an instrument which has a generic yet holistic approach to the assessment. WHO-QoL BREF scale is a cross-culturally comparable, multidimensional, QoL measure that assess it in four domains [13]. This would enable healthcare professionals and health system to help devise relevant interventions to improve the QoL of drug-resistant TB patients and thereby the national programme. Hence our study aims to measure the differential effect of MDR-TB on various domains of QoL and also check for the interaction effect if any.

## Materials and methods

**Figure 1:**
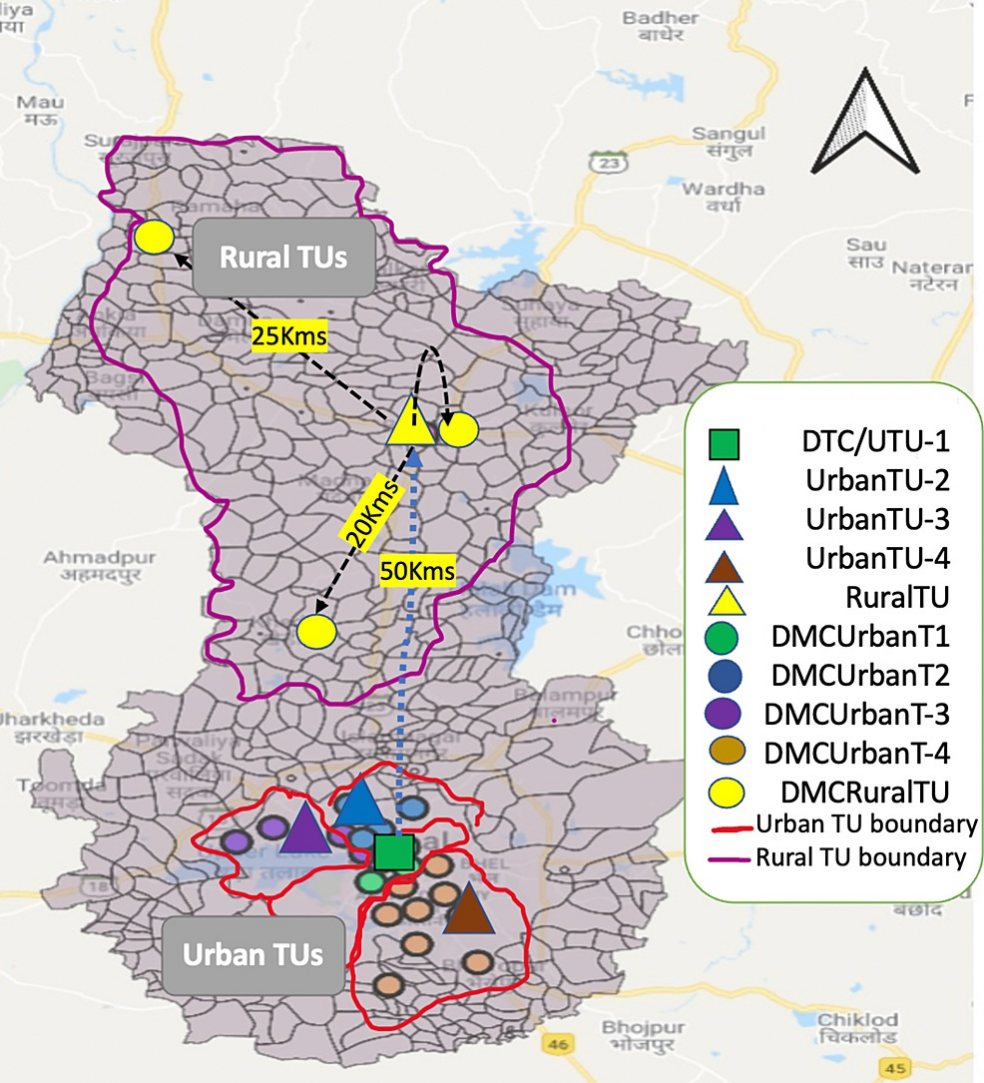
Geographical location of the designated microscopy centres (DMCs) in Bhopal district DTC: district tuberculosis centre; TU: tuberculosis unit; UTU: urban tuberculosis unit

This community-based cross-sectional study was conducted among MDR-TB patients in all the five tuberculosis units (TUs) (4 urban and 1 rural) of Bhopal District. All the patients documented as confirmed microbiological MDR-TB (defined as resistance to both isoniazid and rifampicin, with or without resistance to other anti-TB drugs) confirmed in four quarters of 2017 and registered at District Tuberculosis Centre Bhopal were eligible to participate in the study. The schematic representation of the study area is shown in Figure 1.

Data collection process

**Figure 2:**
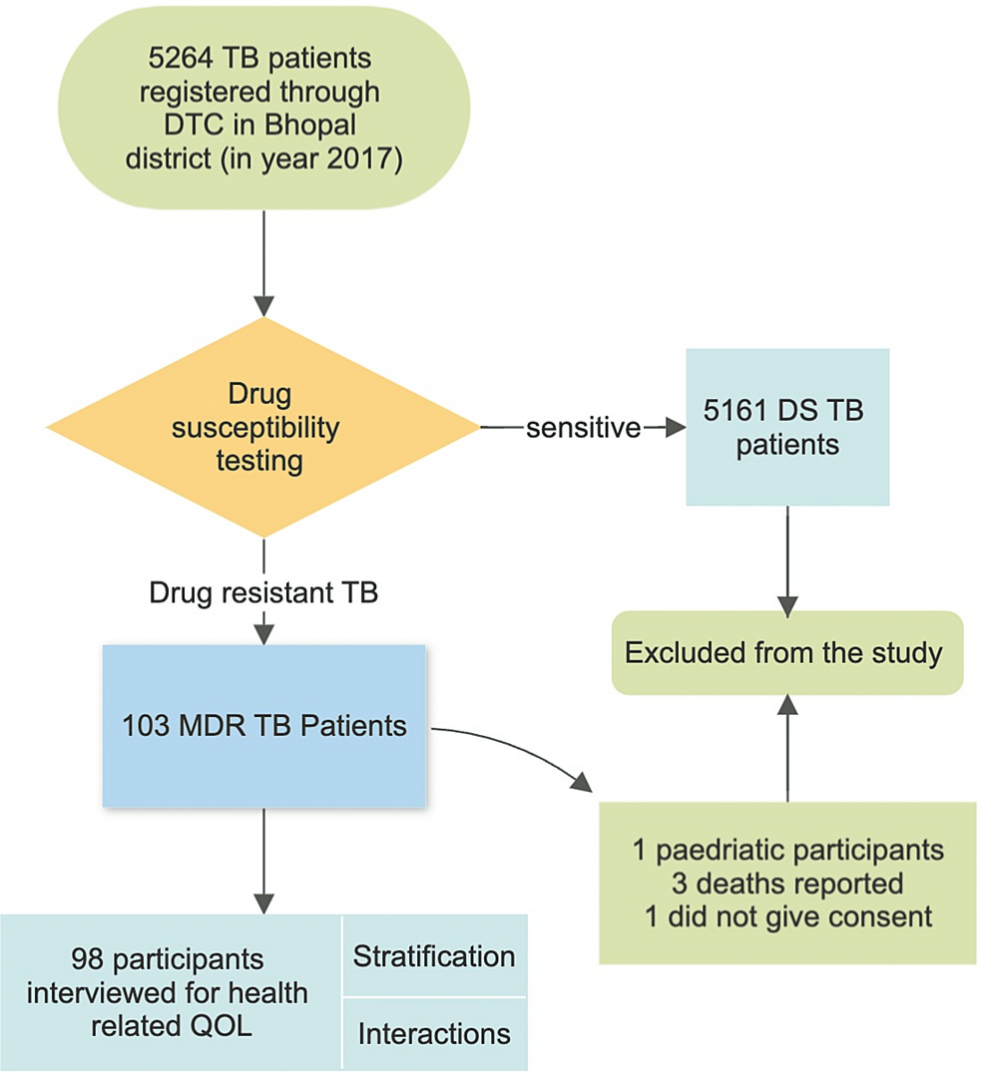
Flowchart showing the inclusion process of participants DTC: district tuberculosis centre; DS TB: drug-sensitive tuberculosis; MDR-TB: Multidrug-resistant tuberculosis; QoL: quality of life

This study had ethical approval from Institutional Human Ethics Committee, AIIMS, Bhopal (vide approval letter no. IHEC-LOP/2018/MD0009). All the potential participants (having age>15 years and residing within the geographical boundaries) of Bhopal district were first attempted contact during their scheduled visit to the centre for medication refilling. In case of patients skipping the scheduled visit, telephonic contact with the help of the Senior Treatment Supervisor was made with the participant to confirm their availability at a place of their convenience. Participants were explained in vernacular about the purpose of the study and further written informed consent was obtained. Patients who were unavailable after two visits on two different occasions or who died or were transferred out while on MDR-TB treatment were excluded from the study. The data was obtained by physical interviews using HRQoL WHO-QoL BREF Scale. The process diagram shown in Figure 2 further quantifies the participation of the patients.

Data collection tool description

The description of the tool for the interested readers is given in supplementary Annexure 1.

The data collection tool consists of two parts. The first section of the tool includes a set of structured questionnaire that detailed the sociodemographic of the patient, including the duration of the treatment, socioeconomic status, type of TB. The second part of the tool was related to assessing the QoL through psychometric analysis. The tool used was the World Health Organisation QoL HRQoL BREF scale. The fact that the WHO-QoL BREF is simple, has universal nature, validated in Indian population and used in varied conditions makes it a good choice for assessing the QoL. We have used the Hindi-validated version of the WHO-QoL BREF Scale.

Statistical analysis

The coded data sheet having the raw score values from each participant was imported to R-environment. It was examined for missing/duplicate values and typographical errors. Recoding of some interval variables was done to categorical variables and information about the time in the continuation phase (CP), socioeconomic status and education was stratified as per used operation definition in the study. This was followed by a descriptive summative domain-wise analysis of transformed score by calculating measures of central tendency (mean/median), measures of dispersions (IQR/SD) and domain-wise item characteristics (floor/ceiling effects). This was followed by exploratory visualisations to understand the transformed scores distributions across the socioeconomic and disease-related characteristics. The extent of agreement for each individual Likert items faceted through domain was also mapped to understand any partisan choices made by participants.

Discriminant validity of the items was checked through the 2-way ANOVA and the post hoc Tukey test amongst the mean (+-SEM) scores in sociodemographic, socioeconomic and disease traits score. We also calculated the interaction effect and the same was also plotted. For statistical purposes, the education of primary level and below were stratified into one category and the rest in the higher education strata. All the continuous variables were expressed in mean with standard deviations, median (IQR) and categorical variables were expressed in proportions. The statistical significance was taken at 0.05 in our study.

## Results

Out of 98 participants, 58 were males, had an overall mean age of 35.13(±14.11) years and all of them were in the CP of treatment. Pulmonary TB was the predominant (80%) TB and around 4/5th of the participants had an urban background. The baseline characteristics of the participants categorised as per treatment duration tertile (early, mid and late CP) are shown in Table 1.

**Table 1:** Baseline characteristics of the participants as per TB treatment duration tertiles ^1^Median (IQR); n (%), CP: continuation phase

Characteristic	Early CP, N = 33^1^	MidCP, N = 33^1^	LateCP, N = 32^1^
Age	38 (28, 54)	27 (21, 40)	30 (26, 36)
Gender			
Female	10 (30.3%)	16 (48%)	14 (43.7%)
Male	23 (69.7%)	17 (52%)	18 (56.3%)
Place			
Rural	8 (24.2%)	7 (21%)	4 (12.5%)
Urban	25 (75.8%)	26 (79%)	28 (87.5%)
Occupation			
Clerical, shop-owner, farmer	8 (24.2%)	1 (3.0%)	0 (0%)
Professional	0 (0%)	1 (3.0%)	1 (3.1%)
Semi-professional	0 (0%)	1 (3.0%)	1 (3.1%)
Semiskilled worker	3 (9.1%)	3 (9.1%)	9 (28.1%)
Skilled worker	4 (12.1%)	0 (0%)	1 (3.1%)
Unemployed	12 (36.3%)	17 (52%)	15 (46.9%)
Unskilled worker	6 (18.1%)	10 (30%)	5 (15.7%)
Education			
Diploma	0 (0%)	0 (0%)	1 (3.1%)
Graduate	5 (15.1%)	3 (9.1%)	8 (25%)
High School	11 (33.3%)	9 (27%)	10 (31.2%)
Illiterate	7 (21.2%)	5 (15%)	4 (12.5%)
Intermediate	5 (15.1%)	7 (21%)	2 (6.2%)
Postgraduate	2 (6%)	4 (12%)	1 (3.1%)
Primary school	3 (9%)	5 (15%)	6 (18.8%)
Household income (INR)	8,000 (5,250, 15,000)	9,000 (6,000, 15,000)	10,000 (6,000, 17,000)
Family H/O TB	27 (79%)	22 (67%)	22 (67%)
Comorbidity present	12 (36.3%)	8 (24%)	5 (15.7%)

Participants had the highest transformed score in the social domain (median score 69, IQR 56-75) followed by the physical domain (median score 41 IQR 31-56) and environmental health domain (median score 38 IQR 25-63). The lowest scores were achieved in the psychological health domain (median 31 IQR 20.5-44).

The respective ceiling percentage for physical domain, psychological domain, social relationship and environmental domain were 0.03, 0.08, 0.25 and 0.08. The floor values were detected as 0.31, 0.22, 0.05 and 0.19 in respective domains. We further calculated the various parameters for the individual items under these four domains. The descriptive statistics consisting of measure of central tendency (mean, median and trimmed mean), measure of dispersion (SD) and distribution characteristics (skewness and kurtosis) are shown in Table 2.

**Table 2:** Descriptive statistic scores of each item of the WHO-QoL-BREF Scale QoL: quality of life

Variable	Mean ± SD	Median (IQR 25%-75%)	Trimmed Mean	Skewness	Kurtosis
Overall QoL	1.98 ±0.93	2.00 (1.00-2.00)	1.84	1.28	1.90
Satisfaction of health	1.96±0.97	2.00(1.00-2.00)	1.82	0.99	0.39
Perceived physical pain	2.11±1.01	2.00(2.00-2.00)	1.95	1.41	1.79
Dependence on medical aid	1.87±0.75	2.00(1.00-2.00)	1.79	1.11	2.65
Positive feeling	1.97±1.18	1.00(1.00-3.00)	1.83	0.79	-0.84
Personal belief	2.48±1.25	2.00(1.00-3.00)	2.39	0.48	-0.88
Ability to concentrate	2.34±1.10	2.00(2.00-3.00)	2.25	0.65	-0.34
Personal safety/security	2.48±1.12	2.00(2.00-3.75)	2.44	0.47	-0.87
Physical environment	2.68±1.27	3.00(2.00-4.00)	2.62	0.16	-1.08
Energy	1.79±0.84	2.00 (1.00-2.00)	1.68	1.16	1.61
Bodily image	2.73±1.10	3.00(2.00-3.75)	2.73	0.02	-0.81
Financial resource/support	1.94±1.17	2.00(1.00-3.00)	1.74	1.12	0.35
Access to information	2.76±1.12	2.00(2.00-4.00)	2.68	0.57	-0.82
Opportunity to leisure activity	2.69±1.41	2.00(1.25-4.00)	2.62	0.30	-1.25
Mobility	2.39±1.12	2.00 (2.00-3.00)	2.30	0.63	-0.39
Sleep/rest satisfaction	2.64±1.22	2.00(2.00-4.00)	2.61	0.25	-1.19
Activity of daily life	1.98±1.00	2.00(1.00-2.00)	1.84	0.99	0.22
Working capacity	1.92±1.10	2.00(1.00-2.00)	1.75	1.07	0.12
Self-esteem	2.63±1.18	2.00(2.00-4.00)	2.61	0.19	-1.12
Personal relationship	3.82±1.07	4.00(3.00-5.00)	3.95	-0.93	0.26
Sexual activity	4.01±0.94	4.00(3.00-5.00)	4.06	-0.48	-0.56
Social support	3.25±1.21	4.00(2.75-4.00)	3.31	-0.52	-0.77
Home environment	2.76±1.25	3.00(2.00-4.00)	2.71	0.10	-1.10
Health care	3.22±1.09	4.00(2.00-4.00)	3.23	-0.29	-1.00
Transport	3.12±1.14	3.00(2.00-4.00)	3.13	-0.18	-0.94
Negative feeling	3.49±1.26	4.00 (2.00-5.00)	3.53	-0.19	-1.37

**Figure 3:**
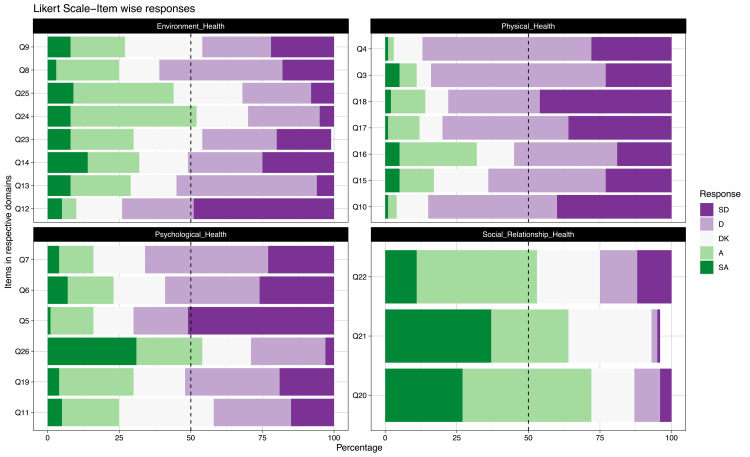
Composite bar graph: item-wise agreement levels with vertical dashed line at median response value SD: strongly disagree; D: disagree; DK: don’t know; A: agree; SA: strongly agree

The visualisation of responses in each items on the Likert scale was created further to understand the extent of agreement with each item in a particular domain (Figure 3). For the ease of understanding, an overall categorical extent of agreement with each item using 50% of participants’ (49 out of 98) response as vertical dashed line is shown in the composite bar chart for each domain. This graph shows an overall achieved higher agreement in the social relationship domain while in physical health domain most of the participants showed dissatisfaction or perceived a lower extent of QoL.

In the physical domain, 50% of the responses showed dissatisfaction in most of the items indicating lesser physical activity level or residual effect of the disease itself. There were very few responses in the items in this domain that had satisfactory notation. On the contrary, in the social domain, 50% of the responses were satisfied with the social support that their family and friends gave during the illness. However, in the environmental and psychological domain, most of the responses recorded did not show any set pattern, there were mixed responses with either a neutral approach or more towards dissatisfaction. The items of the environmental domain that deals with the external determinants of health, also had dissatisfactory expressions in most of them. MDR-TB is not only challenging in terms of medical illness per se but also economic and social implications could be hazardous as evident from the responses recorded.

The distributions of domain-wise score as per personal characteristics, place of residence, socioeconomic status and disease characteristics are shown in the next four tables. Tables 3-4 show no significant statistical interaction of gender and age and place of living in all four domains of QoL. Table 5 shows a statistically significant interaction of education with income tertile evidential more in the physical domain followed by the environmental domain. Table 6 shows a statistically significant effect of type of TB on the psychological domain while the interactive effect of type of TB with the duration of treatment did not show any significant interaction.

**Table 3:** Distributive scores and interaction effect of the domains according to age and gender of the participant Values are means ± SEM. yrs: years, G x A1 = Gender x age category interaction effect

Domain	Female		Male		p-value		
	Age<40 yrs (n=35)	Age>40 yrs (n=5)	Age<40 yrs (n=35)	Age>40 yrs (n=23)	Gender	Age Category	GxA^1^
Physical	44.4±2.26	42.6±6.77	47.1±2.74	41.2±3.71	0.847	0.183	0.638
Psychological	33.3±2.76	45±7.05	35.1±2.73	38.8±3.33	0.603	0.146	0.372
Social	64±3.09	63.8±3.18	67.9±3.66	68.5±4.56	0.315	0.929	0.945
Environmental	40.3±3.26	40.4±6.19	44.9±4.45	42.9±4.37	0.411	0.775	0.867

**Table 4:** Distributive scores and interaction effect of the domains according to age and place of living of the participant P × A^1^ = Place × age category interaction effect

Domain	Rural		Urban		p-value		
	Age<40 yrs (n=15)	Age>40 yrs (n=4)	Age<40 yrs (n=55)	Age>40 yrs (n=24)	Place	Age Category	PxA^1^
Physical	40.5±3.92	47.2±9.47	47.2±1.97	40.5±3.47	0.412	0.192	0.162
Psychological	27.7±3.52	41±1.73	35.9±2.22	39.7±3.49	0.116	0.148	0.34
Social	60±6.02	50±11.6	67.6±2.57	70.6±3.74	0.04	0.824	0.288
Environmental	33.5±5.89	33±5.37	45.1±3.09	44±4.19	0.048	0.846	0.965

**Table 5:** Descriptive scores of the domain among the patients stratified by education status, income tertile and the interaction effect amongst them Values are means ± SEM. ^a-c^Means in a row without a common superscript letter differ (P < 0.05) as analysed by two-way ANOVA and the Tukey test. 1E × I = Edcs × Inct interaction effect. E x I^1^ = Education x income tertile interaction effect Edcs: education; Inct: income tertile Illiterate and primary level education was stratified as E_Strata0 and education above primary level as E_ strata 1. Income of the patients were grouped into tertile by arranging it in ascending order and dividing into three equal divisions.

Domain	E_Strata0			E_Strata1			P-value		
	Tertile1 (n = 17)	Tertile2 (n = 10)	Tertile3 (n = 3)	Tertile1 (n = 15)	Tertile2 (n = 23)	Tertile3 (n = 30)	Edcs	Inct	E×I^1 ^
Physical	39 ± 2.9^bc^	36 ± 2.5^bc^	19 ± 9.5^c^	42 ± 2.6^ac^	47 ± 3.5^ab^	53 ± 2.8^a^	<0.001	0.363	0.008
Psychological	32 ± 3.2	32 ± 5.1	38 ± 11	29 ± 3	35 ± 3.5	43 ± 3.3	0.195	0.029	0.621
Social	58 ± 4.5	63 ± 6.7	75 ± 14	58 ± 4.9	70 ± 3	73 ± 4	0.068	0.027	0.671
Environmental	28 ± 3.2^b^	30 ± 6.3^b^	34± 7.5^ab^	36 ± 3.2^b^	43 ± 4^b^	61 ± 4.3^a^	<0.001	<0.001	0.357

**Table 6:** Descriptive scores of domains among participants stratified by type of TB and duration of treatment (continuation phase) and their interaction effect CP: continuation phase; TxD^1^ = type of tuberculosis x duration of treatment interaction effect

	Extrapulmonary			Pulmonary			p-value		
Domain	EarlyCP (n = 4)	MidCP (n = 3)	LateCP (n = 3)	EarlyCP (n = 29)	MidCP (n = 30)	LateCP (n = 29)	type TB	duration	T×D^1 ^
Physical	47.2 ± 13.9	44 ± 6	58.3 ± 12.5	44.2 ± 2.43	44.9 ± 2.41	43 ± 3.47	0.301	0.994	0.464
Psychological	54.8 ± 12.6	41.7 ± 9.02	44 ± 13.1	31.4 ± 2.6	36.6 ± 2.67	35.6 ± 3.23	0.017	0.694	0.316
Social	79.8 ± 6.9	83.3 ± 9.02	62.3 ± 9.49	69.2 ± 3.06	61.9 ± 3.78	65.1 ± 4.29	0.13	0.386	0.376
Environmental	46.8± 17.8	50 ± 13.1	52.3 ± 21.2	44.5 ± 4.33	42.5 ± 3.65	39 ± 4.06	0.334	0.732	0.835

**Figure 4:**
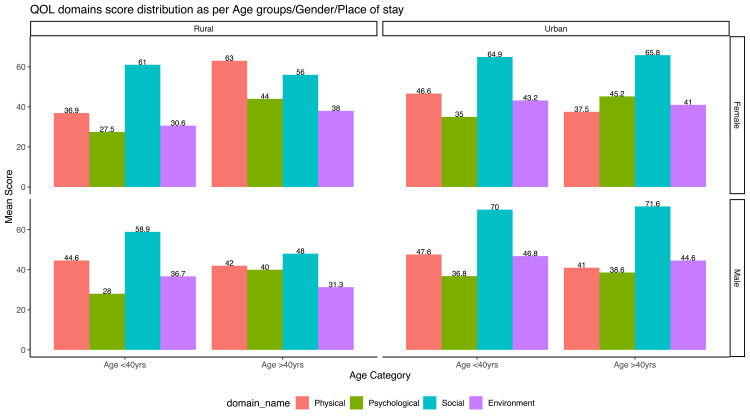
Composite bar graph showing mean transformed score across the age, gender and place of living Overall, the scores obtained were higher in urban males compared to rural females. This visual inequity was more evident in older ages. Psychological health domain scores were secured least across all permutations may indicating a negative encompassing effect of the illness.

**Figure 5:**
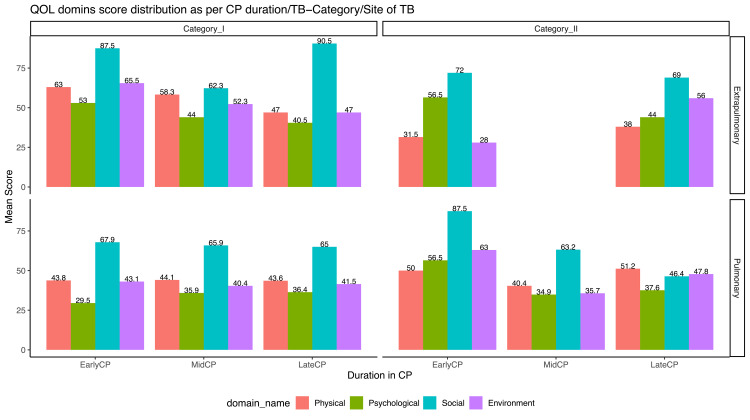
Mean domain scores across the type of TB, time duration from the start of treatment and TB category CP: continuation phase In general, there was a declining trend in perceived QoLs (except social relationship domain) amongst patients with time. These trends seem to be more evident in the newly detected pulmonary TB group compared to extrapulmonary older patients where there are no visible trends.

**Figure 6:**
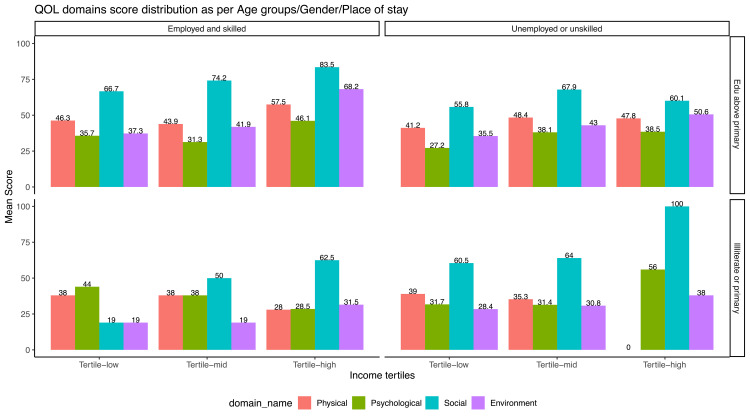
Distribution of domain scores of patients stratified by education status, income tertile and occupation status Unemployed and unskilled labour were unified into a single category. Similarly illiterate and primary level education was clubbed into a single category looking at their background  vulnerability. The income of the participants were arranged into ascending tertiles.

We further looked into the phenomena from a three-dimensional perspective in which QoL domain distribution was stratified across three variables (Figures 4-6).

The educated persons engaged with semiskilled/skilled job perceived a higher QoL compared to their counterparts. This inequity was more visible in the social relationship domain across all strata.

**Figure 7:**
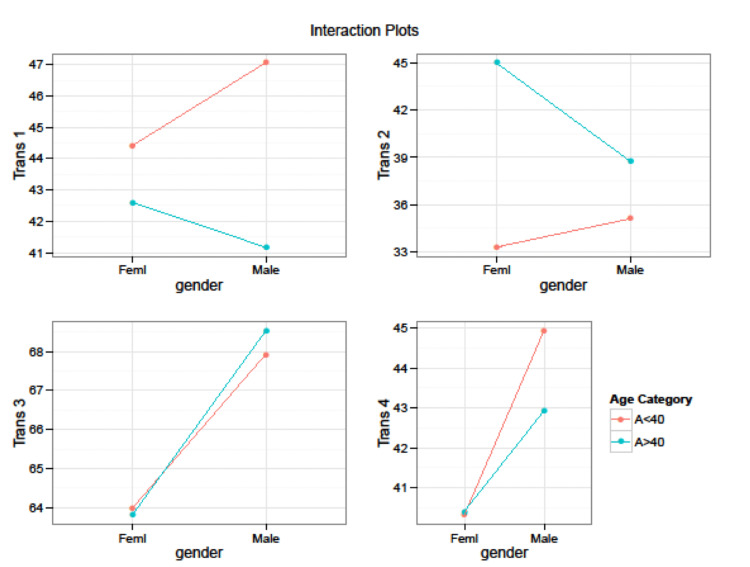
Interaction plot between the gender and age in the four domains Trans1: Transformed score in physical health domain Trans 2: Transformed score in psychological health domain Trans 3: Transformed score in social relationship health domain Trans 4: Transformed score in environmental health domain Feml: Female

**Figure 8:**
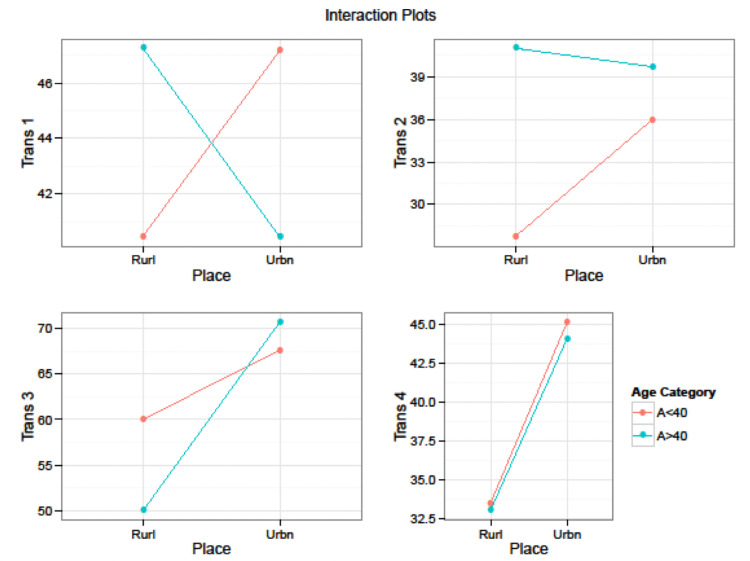
Interaction plot between the place of residence and age in the four domains of HRQoL Trans1: Transformed score in physical health domain Trans 2: Transformed score in psychological health domain Trans 3: Transformed score in social relationship health domain Trans 4: Transformed score in environmental health domain Rurl: rural; Urbn: urban; HRQoL: health-related quality of life

**Figure 9:**
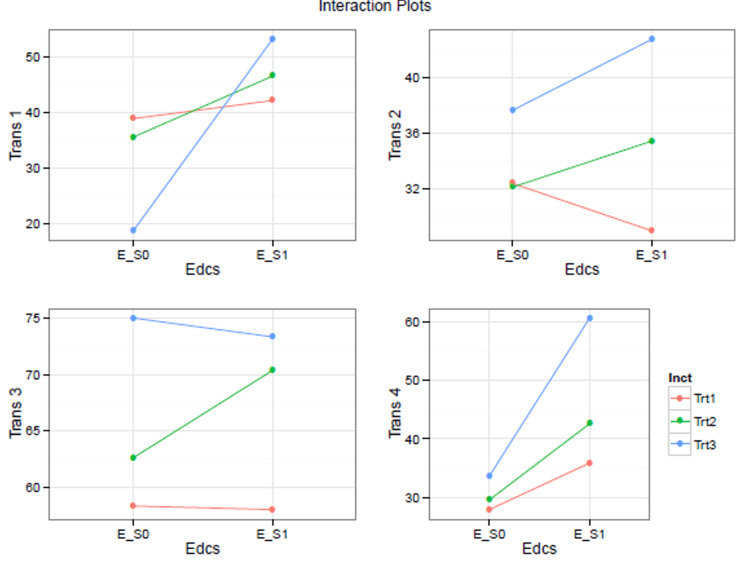
Interaction plots between education status and income tertiles Trans1: Transformed score in physical health domain Trans 2: Transformed score in psychological health domain Trans 3: Transformed score in social relationship health domain Trans 4: Transformed score in environmental health domain Edcs: education strata; E_S0: illiterate and primary level education; E_S1: education above primary level; Trt: income tertile; Trt 1, 2, 3 in ascending order

Some of these interactions were not statistically significant yet a visual exploration showed the nature and direction of the possible interactions effect. These visual interactions between gender and age (Figure 7), place of residence and age (Figure 8) and educational status and income tertiles (Figure 9) are shown in the supplementary annexure.

## Discussion

QoL is a relatively speculative construct having different meanings in different contexts. An inclusive objective definition of QoL is thus difficult to draw. Often it is equated with the subjective perceptions of an individual pertaining to his aspirations, belief system, concerns and perceptual extent of fulfilment. These individualistic opinions are further highly influenced by cultural and macro-environmental contexts [14]. Thus QoL by principle seems to be a multidimensional paradigm which has to be viewed and measured from a holistic perspective. The dominion inter-dependence also corroborates with the arguments which can be seen in the present study. The WHO-QoL BREF in this background offers an opportunity to look into the phenomenon through a cross-cultural perspective, across the disease and can be utilized in intra-disease comparison.

Distribution of score

Overall QoL scores seem to be tilted in favour of an urban setting, male gender and towards a relatively younger population [15]. The exception to this axiom can be seen in a psychological domain where the relatively older population performed better. As, with age, people in general become firm psychologically, accommodating, emotionally settled yet physically less active which is reflected in the scores [16]. Similar results were reported in the study by Aggarwal et al. where they found that female scored lower than male and had a negative impact on regression analysis on all baseline domain scores obtained at the initial treatment phase [8]. In coherence with our result, Meera Dhuria et al. reported females having lower mean scores (11.67 ± 1.26) than males (11.85 ± 1.66) for overall QoL however they fared better in the physical and environmental domain [17]. Gender inequality in the Indian context has generally perceived females as the weaker sex thereby having poor health-seeking behaviour and tend to avoid treatment and care unless the disease advances [10,18]. This highlights the need for necessary support that can facilitate females for a better and self-sufficient life. Muniyandi et al in their study did not find any gender difference in physical domain but had lower scores in mental and social wellbeing [19].

In early to late CP of treatment there was a gradual decline in three out of four domains amongst the new pulmonary TB patients. A reasonable explanation may be the requirement of polypharmacy for a longer duration and the inability to cope up with indirect costs involved in the disease [20]. Also due to the high chronicity and detrimental nature of the disease, residual impairment in HRQoL is quite an unfavourable outcome even after the treatment [21]. However, mathematically this can be another example of random variations after splitting a major group into several small subgroups. More educated participants engaged in the semiskilled and skilled job were found to be less vulnerable compared to their counterparts and this difference visually remains there at large after stratifying with income. This may indicate the perceived need for information and service accession may be fulfilled by education and that converts a tense individual into a contented person [22]. Similarly, another study by Malik et al. reported participants with intermediate or higher education to have better mental component score as compared to illiterate patients [16].

The physical dimensions of the QoL were hit hardest by the disease and the evident or palpable items (pain and discomfort, mobility, and activity of daily living etc.) were perceived to be affected more by the disease [23]. This alleged deficit in physical health and consequent deviation from the title role in society seems to affect the psyche of an individual in terms of his inability to enjoy, perceiving meaning in life and inability to concentrate which is evident from this study [24]. Participants have shown relative satisfaction with the interpersonal relationship with immediate accomplices like spouses and close friends compared to other domains which may be explained more by the construct of questions than the socio-cultural context. Similarly, items on personal relationships and sexual activity, seem very personalized in nature. The other explanation for achieving greater agreement in this domain is definitely related to strong ties and family values in the Indian socio-cultural context.

However a specific adversarial event like TB may have its due weightage to influence QoL negatively. A major disease (like in this study) has its own emotional, medical, structural (social), health behavioural, psychological, functional and economic effects on QoL on the one hand which may be summarized as intrinsic or inherent effects [25]. Coupled with these intrinsic effects, the macro-environmental customs, beliefs, discriminations and stigma associated warrant specific rather generic QoL measures for diseases like TB [26].

QoL in MDR-TB may be perceived as a proxy marker of the extent of satisfaction with mutually agreed upon decisions (adherence ) between program and patient apart from patient-specific intrinsic factors and this may further influence the prognosis at the individual level and treatment outcome at the programmatic level [9]. Thus at the policy level, programs should be sensitive not only to formulations/implementations of the clinical dimensions but also to the socio-cultural, psychosocial and economic context. All attempts are thus to be directed more towards vulnerable sections in terms of provision of enablers and counsellors specifically to the extreme end of the disease spectrum like MDR and XDR-TB. The development of a psychometrically robust TB-specific tool in the Indian context thus may offer an objective tool to measure the extent of achieving these aspirations in NTEP.

This study to the best of our knowledge is one of the pioneers to understand the QoLs concerns in patients with MDR-TB in a native setting. It contemplates the phenomenon through the possible interactions with other covariates. The study attempts to cover all geographical settings and socioeconomic groups which are likely indicative of areas of QoL affected by the illness. However since the study was conducted in DR-TB patients of a specific district, its representativeness to the entire population of the state or country may be a concern. The resultant seemingly small sample size should be seen in the context of the relative rarity of the phenomena, stringent inclusion criteria and attempt to restrict in a reasonably homogenous geo-environmental setting. Another limitation seems to be the inability to measure the test-retest validity due to the cross-sectional nature of the study yet we tried to overcome this by analysing the marginal effect of different timing in CP on QoL.

## Conclusions

MDR-TB itself and its known risk factors have a significant encompassing effect on the HRQoL of patients. Different spheres of life are affected differently and so it has its unwarranted repercussions. The additional parameter of periodical QoL assessment apart from the regular clinical and microbiological tests can thus not only help in evaluating the efficacy of the treatment but also help in timely and appropriate action by the health care providers of NTEP.

## Figures and Tables

**Figure 10:**
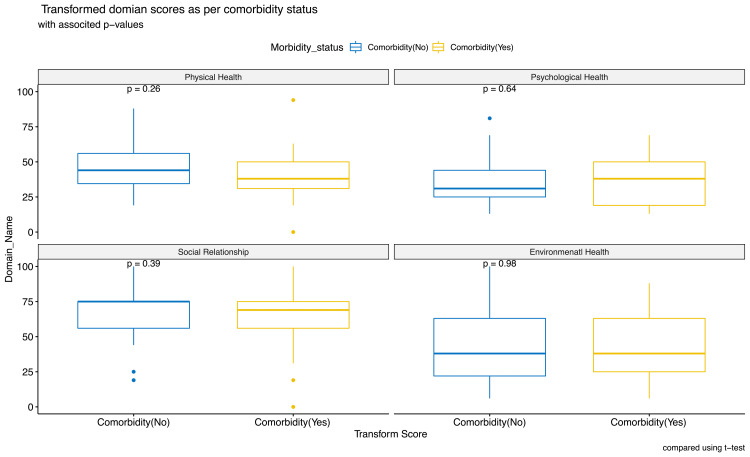
Box-plot showing transformed scores faceted by comorbidity status A statistically nonsignificant association of QoL scores with comorbidity status was detected in all domains. 25/100 participants had some comorbid conditions. The most common comorbidities were hypertension (n = 14) followed by diabetes (n = 12).

## Appendices

Supplementary annexure 1

Tool Description

The scale consists of 26 questions, essentially a construct of dimensions that influences an individual’s physical, mental and social wellbeing. Twenty-four of 26 items measure the HRQoL in four domains namely physical, environmental, social and psychological domains. Two other items measure the overall QoL and general health. The scoring is done under four dimensions physical, environmental, social and psychological domains. Each item is rated on a 5-point Likert scale from the lowest score as 1 to the highest score of 5. The mean score is computed to form the raw score of each domain. The raw score is then multiplied by 4 to transform the score into a scaled score which is then compared with the original WHO-QoL 100. The QoL score in each dimension is graded on 0 - 100 scale. The higher the mean scores of the domain, the better the QoL in that domain. The physical health domain incorporates seven facets of activities of daily living, dependence on medicinal substances and medical aid, energy and fatigue, mobility, pain and discomfort, sleep and rest, and work capacity. The psychological domain assesses six facets related to body image and appearance negative feelings, positive feelings, self-esteem, spirituality/religious/personal beliefs, thinking, learning, memory and concentration. The social relationship domain has three items that assess personal relationships, social support and sexual activities. While environmental domain assesses eight facets for financial resources, freedom/physical safety/security, accessibility and quality of health and social care, home environment, an opportunity for acquiring new information and skills, participation in and opportunities for recreation and leisure activities, physical environment  (pollution/noise/traffic/climate) and transport.

Overall, the female patients scored low in all the domains as compared to the male and in respective age groups. In our study higher age groups were observed to score better in the psychological and social domains of both genders may be because of better coping mechanisms in dealing with varied challenges of life. The social networking and associations and greater disposition to spirituality in higher age group especially in the Indian context have also led to better QoL as substantiated with scores. The HRQoL scores however had a decline in the physical domain and environmental domain with age. This could have been explained by the fact that with increasing age as the physical endurance decreases so does the working capacity and financial security. The changes in the scores observed however did not have statistical significance. The same could be observed in the interaction plot wherein the social domain, and the crossed lines suggest the interaction effect among the two variables, though they are not statistically significant (p-value 0.945).

In the urban sector, the younger participants scored higher in the physical and environmental domains. Interaction effects are seen in the physical health domain and social domain (Table 4). The figure shows that age and place of residence have interaction effects on the physical health and social domain. Younger participants tend to have better physical work capacity if they reside in the urban sector though the same was not statistically significant (p-value 0.162). Another interaction effect could also be seen in the social domain, where patients have social support irrespective of age in urban.

The HRQoL scores are better in the participants with higher education status. As the income increase the domain scores also increased especially in the psychological, social and environmental domains. The interaction effect is statistically significant in the physical health domain which can also be visualised in the figure. This can be explained by the fact that education leads to better employment opportunities and improved socio-cultural factors. Economic security also contributes to psychological and social security that motivates for better living and health-seeking behaviour. The interaction plot between the income tertiles and the education status shows that HRQoL physical domain scores are higher if the education is high especially in participants in higher income tertiles. This was further confirmed by the interaction effect with a statistically significant p-value of 0.008 in the physical domain (Table 6).
